# Community Hospitals in Selected High Income Countries: A Scoping Review of Approaches and Models

**DOI:** 10.5334/ijic.2463

**Published:** 2016-11-24

**Authors:** Eleanor M. Winpenny, Jennie Corbett, Celine Miani, Sarah King, Emma Pitchforth, Tom Ling, Edwin van Teijlingen, Ellen Nolte

**Affiliations:** 1 RAND Europe, Westbrook Centre, Milton Road, Cambridge, CB4 1YG, United Kingdom; 2 Bournemouth House B112c, 19 Christchurch Road, Bournemouth, BH1 3LH, United Kingdom; 3 London School of Economics and Political Science, Houghton Street, London WC2A 2AE, United Kingdom

**Keywords:** cottage hospital, primary care, health systems, service delivery models, integration, primary/secondary care interface, community hospital

## Abstract

**Background:**

There is no single definition of a community hospital in the UK, despite its long history. We sought to understand the nature and scope of service provision in community hospitals, within the UK and other high-income countries.

**Methods:**

We undertook a scoping review of literature on community hospitals published from 2005 to 2014. Data were extracted on features of the hospital model and the services provided, with results presented as a narrative synthesis.

**Results:**

75 studies were included from ten countries. Community hospitals provide a wide range of services, with wide diversity of provision appearing to reflect local needs. Community hospitals are staffed by a mixture of general practitioners (GPs), nurses, allied health professionals and healthcare assistants. We found many examples of collaborative working arrangements between community hospitals and other health care organisations, including colocation of services, shared workforce with primary care and close collaboration with acute specialists.

**Conclusions:**

Community hospitals are able to provide a diverse range of services, responding to geographical and health system contexts. Their collaborative nature may be particularly important in the design of future models of care delivery, where emphasis is placed on integration of care with a key focus on patient-centred care.

## Key policy messages

- Community hospitals cover a diverse range of services in their communities; this makes it important for policy-makers to be clear about what they mean when they talk about community hospitals.
- Community hospitals can perform an integrative role in local service provision, through close working with primary care services and with acute hospitals, which may be particularly important in the design of future models of care delivery.
- New technologies including telemedicine and videoconferencing can enhance the capability of community hospitals to deliver an increasingly diverse range of services in the community, supported by specialist providers.
- There is considerable potential for cross-country learning to help improve health service delivery.
- Further evidence is required on the (cost-) effectiveness of delivery of particular services in community hospitals compared with other locations.

## Introduction

Community hospitals have been an established component of health care provision in the United Kingdom (UK) for many decades, frequently evolving from small ‘cottage’ hospitals, which predated the formation of the National Health Service (NHS) in 1948 [1]. Community hospitals have typically been staffed by mainly general practitioners (GPs) and nurses to provide care in a hospital setting, often for predominantly rural populations [2]. They usually sit at the interface between primary and secondary care [3] and may provide a diverse range of services including inpatient, outpatient, diagnostic, day care, primary care and outreach services [1].

In England there has been increasing policy focus and government investment into shifting the delivery of medical care to community settings [4,5], with calls for the development of a new generation of community hospitals and services that would be responsive to local needs and at the forefront of health care innovation [6,7,8]. The 2014 NHS Five Year Forward View proposed new models of care to be developed in England which allow for integration across organisational boundaries, and highlighted the potential role for community hospitals in delivering more integrated care locally by bringing together community, primary and secondary care services [9]. Similar visions have been expressed in other system contexts [10,11,12]. While there is potential for community hospitals to assume a more strategic role in service delivery, the precise role these service structures should take is not clear.

Community hospital have been viewed in different ways, as stepdown facilities, as an extension of primary care, or as an alternative to secondary care [1]. A wider range of service delivery arrangements have also been described, such as community care resource centres, community care homes and intermediate care or rehabilitation units [6,7,13,14]. There is however, a need to better understand the different roles community hospitals can fulfil, and their capacity and capability to integrate or collaborate with other health and care services. This seems to be of particular relevance in the English context, where a small number of so-called ‘vanguard’ sites for new care models have included community hospitals as part of integrated primary and acute care systems and multispecialty community providers [15]. At the same time, other community hospitals have been closed or face closure [16], suggesting that their strategic positioning within the NHS remains unclear.

Commissioned by the National Institute for Health Research (England), this scoping review updates a review by Heaney and colleagues published in 2006 [1]. It aims to describe different models of community hospitals in selected high income countries. From this understanding of the nature and scope of services provided and their specific functions we seek to inform the future development of community hospitals.

## Methods

We carried out a scoping review [17] following the approach proposed by Levac et al. [18]. Given the broad range of descriptions regarding what constitutes a community hospital (see Table 1), we developed a working definition to guide our review. After reviewing these definitions, and having sought expert opinion from members of our project advisory board we stipulated that a community hospital (i) provides a range of services to a local community, (ii) is led by community-based health professionals, and (iii) provides inpatient beds.

**Table 1:** Definitions of ‘community hospital’.

Definition	Reference

A general practitioner community hospital can be defined as a hospital where the admission, care and discharge of patients is under the direct control of a general practitioner who is paid for this service through a bed fund, or its equivalent.	Liaison Group of the Royal College of General Practitioners the Association of General Practitioner Community Hospitals Royal College of General Practitioners (1990) [2]
A community hospital is a local hospital, unit or centre providing an appropriate range and format of accessible health care facilities and resources. Medical care is normally led by GPs, in liaison with consultant, nursing and allied health professional colleagues as necessary and may also incorporate consultant long stay beds, primary care nurse-led and midwife services.	Ritchie (1996) [80]
Many countries have a lower tier of hospital, sometimes called a community hospital. These typically have 50 beds or less and provide basic diagnostic services, minor surgery and care for patients who need nursing care but not the facilities of a district general hospital.	McKee and Healy (2002) [81]
A service which offers integrated health and social care and is supported by community-based professionals.	UK Department of Health (2006) [4]
A local hospital, unit or centre community based, providing an appropriate range and format of accessible health care facilities and resources. These will include inpatient beds and may include outpatients, diagnostics, surgery, day care, nurse led, maternity, primary care and outreach services for patients provided by multidisciplinary teams.	Community Hospitals Association (2008) [78]

We searched PubMed, Embase, Scopus, CINAHL, PsycINFO, British Nursing Index, HMIC, Social Care Online, and Health Business Elite in June 2014 for literature published since 2005, using the principal search terms “community hospital”, “cottage hospital”, “GP beds” or “intermediate care”. The full PubMed search strategy, which was adapted for the other databases we used, is shown in Additional file 1.

Three researchers (CM, SK, JC) screened titles and abstracts of identified records against a set of inclusion and exclusion criteria (Table 2), which were informed by our working definition. The researchers independently screened the same 300 records and compared their results in order to ensure consistency in deciding on study eligibility. The remaining titles and abstracts were then screened by one of the three researchers. Full texts of potentially eligible studies were retrieved and re-assessed against the inclusion and exclusion criteria by one reviewer, and checked by a second reviewer. Disagreements or uncertainties between reviewers were resolved by discussion within the wider research team.

**Table 2:** Inclusion and exclusion criteria.

	Inclusion criteria	Exclusion criteria

**Setting**	High income country with comparable health care systems that provide universal access (ie Canada, Australia, NZ and high income countries in Europe)	Low- and middle- income country ; non-European country (except Canada, Australia, NZ)
**Facility type**	Meets all of the following criteria: Provides beds Is led by community-based health professionals Provides a range of services to a local community.	Facility that offers specialist services only GP- or nurse-led beds within secondary or tertiary hospitals
**Outcomes**	A description of the nature and scope of delivery models or services provided	Provides synthesis and discussion of the delivery model only Does not describe the delivery model or services provided by individual community hospitals
**Study type**	Experimental study (randomised control study (RCT), cluster-randomised controlled trial, quasi-randomised controlled trial), and observational study	Editorial, commentary, review
**Publication type**	Journal article, report, dissertation, book and professional journal	Conference abstract, study protocol
**Publication year**	Published in 2005 and after	Published before 2005
**Language**	All languages	n/a

Data were extracted from each study on the features of the hospital model (e.g. management, staffing, ownership) and the specific services offered. Data extraction was undertaken by the three researchers with some duplicate extraction to check for consistency of the approach. Data were analysed drawing on the principles of narrative synthesis, which has been recommended as the most appropriate approach for analysing diverse evidence [19].

Given this diversity, it was not possible to conduct a formal quality assessment of the included studies. However, during data extraction researchers commented on the nature of the evidence and noted any concerns about study quality. Ethical approval was granted by Bournemouth University; no further NHS research ethics permission was required as this review does not involve primary research with human participants.

## Results

**Figure 1:**
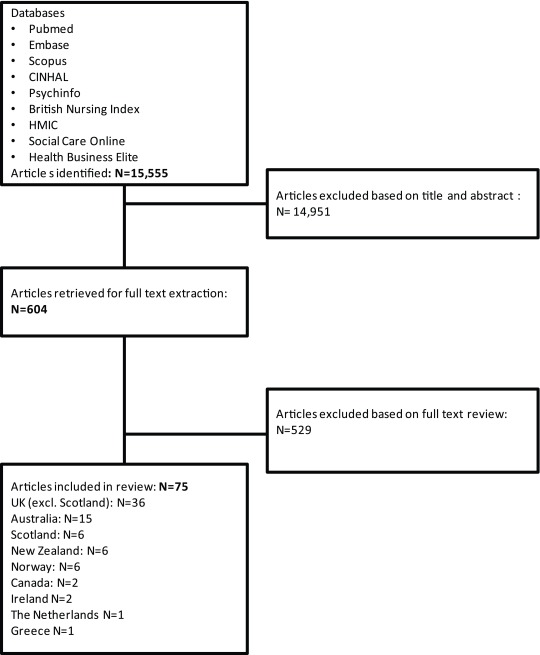
Flow chart depicting literature search and exclusion process.

Our searches identified a total of 15,555 records following the removal of duplicates. After initial screening of titles and abstracts, we considered 604 references for full text review, and of these, 75 studies were identified as eligible for inclusion (Figure 1).

The majority of studies were descriptive or used an observational design, while 11 studies used a randomised controlled trial (RCT) design. Included studies fell broadly into the following categories: descriptions of one or more community hospitals (n = 14), descriptions of development of new facilities or procedures within a community hospital (n = 9), reports of particular services within community hospitals (n = 12), studies of patients’ or family members’ experiences of care within community hospitals (n = 4), studies presenting surveys of community hospitals or units within community hospitals (n = 5), or studies reporting on specific outcomes of care delivered by community hospitals (n = 9).

The largest number of studies were set in England and Wales (n = 36), followed by Australia (15), New Zealand (NZ) (6), Norway (6), Scotland (6), Canada (2), Ireland (2), the Netherlands (1) and Greece (1).

Table 3 provides an overview of selected data from eligible studies, including the range of services provided by the community hospitals, as well as the types of staff involved in delivering the services. It should be noted that for many of the studies found, reporting of details of the hospital model was not the primary aim, and information presented here was taken from background or introductory information provided.

**Table 3:** Overview of services provided by community hospitals in different countries.

Country	Number of papers retrieved	Facility designation	Services discussed in the literature	Staffing

England and Wales	36	Community hospital	A large proportion of articles focused on non-acute inpatient services e.g. post-acute care, rehabilitation or palliative care. Fewer articles looked at outpatient services, urgent care such as in minor injury units, and acute inpatient care. Other services that were discussed more rarely include health promotion, surgery, mental health care, primary care, social care and maternity care	Care led by GPs, nurses and/or community geriatricians, supported by specialist consultants and other practitioners
Scotland	6	Community hospital	Articles reported on non-acute inpatient services, outpatient services, urgent care services, acute inpatient care, surgery, mental health care and maternity care.	Not reported
Norway	6	Intermediate care hospitalCommunity hospital	All articles discussed provision of non-acute inpatient services, particularly intermediate care. Other services included outpatient services, urgent care services, acute inpatient care, mental health care and maternity care.	GPs, nurses and allied health professionals
New Zealand	6	Rural hospital	Articles reported on the provision of non-acute inpatient services, outpatient services, urgent care services, acute inpatient care, surgery, and primary care	GPs, Medical Officers of Special Scale, nurses and allied health professionals. Visiting specialists
Australia	15	Rural hospitalRegional hospitalBase hospital	Articles reported on the provision of non-acute inpatient services, outpatient services, urgent care services, acute inpatient care, surgery, and primary care	GPs, nurses, midwives and allied health professionals
Canada	2	Rural hospital	Articles report on provision of acute and non-acute inpatient care, urgent care services, surgery, mental health care and maternity care	Family physicians
Greece	1	Hospital-health centre	The article reports on provision of inpatient, outpatient, primary care and preventative health services	Doctors and nurses
Ireland	2	Community hospital	The articles report on provision of non-acute inpatient services and outpatient services	Nurses and allied health professionals, with input from GPs and geriatricians
The Netherlands	1	General practitioner hospital	The article reports on provision of acute and non-acute inpatient care, outpatient services.	GPs and nurses with support from paramedics and specialists

### Range of services provided by community hospitals

Community hospitals provide a wide range of services, covering the whole spectrum of care provision, from preventative [20,21] and primary care [22,23], through to outpatient services [24,25,26], inpatient medical care [27,28], surgery [29,30], minor injury [31] and accident and emergency care [22,32]. Within these broad areas, there was considerable diversity of the types of services provided, and a number of studies further reported on the implementation of new and innovative types and methods of service provision not previously available within the community hospital setting, such as point-of-care testing [23], fracture clinics [33] or chemotherapy [34].

Community hospitals that provided a wide range of services were common in Australia, NZ and Canada, reflecting the geographical needs of these countries in ensuring provision of locally accessible primary, secondary, and emergency care services in remote rural areas. However, providing comprehensive services in these settings was reported to be challenging, because of limited capacity or access to specialist expertise to deliver the services required to meet the needs of the local population. For example, one study set in NZ reported that of 35 selected medical conditions and procedures that may be needed for acutely ill patients, only about 70% could be performed in any one of a group of rural hospitals [35]. A cross-sectional survey of emergency departments in rural hospitals in Canada found that, with the exception of basic laboratory and x-ray services, the majority had limited access to professional and support services. For example, only 5% of hospitals had access to a paediatrician, 26% to a surgeon and less than one third had access to ultrasound equipment (28%), a CT scanner (20%) or an intensive care unit (17%) [36].

Studies of community hospitals in England and Scotland typically reported on the provision of non-acute inpatient services, particularly post-acute geriatric care, rehabilitation services and palliative care [37,38,39,40,41,42]. Indeed, several UK community hospitals provide exclusively, or largely, non-acute inpatient care to chronically ill or older populations [20,38,40]. Similarly, community hospitals in Ireland tend to focus on services for older people such as respite care, rehabilitation, palliative care long-stay facilities and community-based assessment [43,44].

Studies of community hospitals in Norway also described a focus on intermediate care, targeted at people who would otherwise face unnecessarily prolonged hospital stays or inappropriate admission to acute inpatient care, including chronically ill and older patients [45,46,47]. A specific case of a community hospital is Hallingdal Sjukestugu in central Norway [27,48]. Described as a ‘decentralised specialist health care service’, it is led by general practitioners under telephone supervision of hospital specialists who are located in an acute hospital which is 170 km away and administers and funds the community hospital. It includes an inpatient department, which functions as an intermediate care unit, along with outpatient psychiatric and somatic services, somatic daycare, a somatic inpatient department, as well as a pre-hospital ambulance and air ambulance services.

There were only a small number of studies of community hospitals in countries other than England, Scotland, Australia, NZ, and Norway, as noted earlier. One study set in the Netherlands described a community hospital that was established as an experiment after the closure of a former district general hospital west of Amsterdam [49]. Its 20 beds were designated as either ‘GP beds’ for GPs treating their own patients, ‘recovery beds’ for the rehabilitation of post-surgery patients, or ‘nursing home beds’ for patients awaiting a place in a nursing home. Services reported were low level care and observation, and included diagnostic facilities (e.g. laboratory and x-ray), allied health services (e.g. physiotherapy, occupational therapy and speech therapy), and outpatient clinics.

A number of studies described and evaluated the development of new outpatient services in community hospitals. Examples include a treatment and diagnostic centre for gynaecology [50] and a nurse-consultant-led clinic for patients with chronic musculo-skeletal pain [24], both set in the UK. Several studies reported on outpatient services that were developed in collaboration with larger hospitals, such as services for people with eating disorders, which used videoconferencing to connect participants in community hospital sites with a specialist service within a large urban hospital for weekly therapy sessions [51]. Another example was the development of a teleopthamology service by a regional hospital in Western Australia together with eye specialists in Perth which allowed digital images to be transmitted to the specialists for diagnosis [26]. Two studies described outreach chemotherapy services, delivering chemotherapy cycles in community hospitals by staff based at a larger hospital or care centre [34,52].

Finally, two studies described the role of community hospitals in the provision of maternity services. One Australian study reported on a rural community hospital providing pregnant women with access to monthly ultrasound, specialist maternity advice by telephone, and an obstetrician outpatient clinic several times a year [53]. Another study, also set in Australia, described a midwifery-led model of care within a rural hospital, providing low-risk women the option to give birth at their local hospital [54].

### Staffing

The community hospital workforce includes GPs, generalist and specialist nurses, allied health professionals (e.g. physiotherapists, occupational therapists, dieticians) and healthcare assistants. This staff mix is described in studies of community hospitals in the UK [40], Norway [45], the Netherlands [49] and Australia [55]. In many hospitals GPs are in charge of hospital management [56], or have ultimate responsibility for patients and beds [49,57]. In NZ the community hospital workforce also includes the non-specialist Medical Officers of Special Scale (MOSSes) [35,58], a non-training position for a doctor who has not yet specialised [59]. Staffing models were described where MOSSes constitute the core of the medical workforce, supported by nursing staff and allied health professionals, together with back-up GPs or visiting specialists [58,60].

In many community hospitals, medical doctors were reported to represent a small proportion of its staff, and were not available on site at all times. For example, a survey of NZ rural hospitals reported that 14% of hospitals had a GP on-site at all times and 41% had a facility for the GP to spend the night in the hospital [35], while a study of the ten community hospitals of the Powys region in Wales noted that none of these had resident medical doctors, including GPs [57]. Elsewhere, studies reported on-site availability of GPs only during weekdays, such as in a 12-bed intermediate care hospital in Norway [45], however, GPs are generally available to provide care at night and during week-ends, with on-call GPs committed to provide out-of-hours cover [23,57,60,61].

In some countries, shortage of medical staff is reported to be an issue. This was the case for NZ, where 9% of medical staff positions were unfilled and 24% filled by locums [62], and in Greece [21]. One study in Australia [54] described difficulties experienced by a rural hospital in recruiting sufficiently skilled hospital medical officers, eventually leading to the closure of the maternity service. However, in this particular case it was possible to substitute medical officers with a midwife, permitting re-opening of the service six weeks after its initial closure.

#### The role of specialists

Given that many community hospitals included in this review do not tend to have GPs on-site full time it is perhaps not surprising that on-site presence of specialists is even less common. In most cases the specialist tends to perform an intermittent or remote supervisory role [43,54]. Models of such supervision include weekly oversight by a consultant from the nearest acute hospital [37], or regular educational visits [43]. One study from NZ reported on consultant surgeons who undertake visits to community hospitals over a distance of 150 km at least twice per week [58]. In more remote areas, specialist visits may be less frequent, such as, in Australia, the delivery of obstetrician outpatient clinics several times per year [53], or of specialist eye care offered by visiting specialists for one week two times per year [26].

This limited or remote specialist involvement means that GPs and nurses are required to be flexible in their roles and to demonstrate a broad spectrum of skills [23,27]. For example, GPs may perform minor surgery or caesarean sections [54] and have “multiple roles”, which include ward duty, GP clinics and emergency unit on-call, such as in a 20-bed rural hospital in Victoria, Australia [63]. Small regional hospitals in the Northern Territory in Australia are staffed with GPs trained to perform emergency and elective surgery [30]. As for nurses, they may have to demonstrate skills in areas such as clinical procedures, diagnosis, leadership, patient-centred care, inter-professional communication, spiritual guidance and bereavement support [64], or to master some relatively complex diagnostic tools (e.g. for stroke [37] or chest pain [31]).

#### The role of nurses

The importance of the nurse’s role was particularly emphasised in community hospitals where, in addition to requirements for a broader skill set, they hold greater managerial [43] and patient-related responsibility than in larger hospitals [65]. Senior nurses or midwifes are often in charge of managing a unit or the whole hospital, as in the case of the 18 community hospitals reported on in Ireland [43]. They may be responsible for the patient from admission to discharge [66], without the patient seeing a doctor [63]. In other cases, nurses were in charge of the development and implementation of a specific specialist service, such as a chronic musculo-skeletal pain service [24] or a mental health liaison service [67]. Steers et al. [68], based on a review of the evidence of providing palliative care in community hospitals in the UK concluded that GPs generally acknowledged their dependence on nursing staff to support them to make timely management decisions following the admission of patients.

### Collaboration and integration with other services

Community hospitals tend to be highly collaborative and integrated with primary and secondary care as well as with third sector or community organisations [69]. This is facilitated through the community hospitals’ role along the patient pathway, its function as a physical site for the co-location of services, and through a shared workforce with primary care and close collaborative working with acute specialists, described above.

For example, one of the functions community hospitals may take on is the provision of post-acute care. A study of the effect of an intermediate care hospital in Central Norway on the discharge process from acute to community care found that the community hospital had a role in facilitating integration between care levels [45]. Staff at the acute hospital saw the community hospital providing ‘an extension of a hospital department’ while those in primary care viewed it ‘as a buffer that provided preparations for discharge of the patients’. Staff of the community hospital appear to liaise effectively with both acute and primary care, sharing information through medical records as well as further direct communication where necessary.

Physical co-location of different services also offers opportunity for collaboration and integration. Included studies report co-location of primary care, community care and social care services within the community hospital [20,70,71]. A perhaps unusual case is that of a community hospital in Oxford, England, which was transferred to form a unit within a large tertiary teaching hospital. Special financial arrangements (a monthly fee) allows staff from the acute hospital, such as the specialist gerontologist, senior registrar and senior house officer, to support community hospital staff [40].

Given the core involvement of GPs in the delivery of community hospital services as described above, typically working in their practice in addition to delivering shifts in the hospital, they provide opportunity to build strong links between the community hospital and primary care [35,49]. Indeed, in the UK, continuity of care delivered by local GPs known to the patient and their family was cited as one of the benefits of care in a community hospital [28].

Strong collaboration was reported between community hospitals and specialists located in acute hospitals, and a number of studies described different models of collaboration. For example, specialists from a nearby acute hospital are frequently reported to be available to provide remote advice and support when needed, for example by telephone or videoconferencing [66]. We earlier described Hallingdal Sjukestugu in Norway, which is funded and administered by an acute hospital with patients legally under the acute hospital’s professional responsibility [27]. As such the GPs are under remote supervision from a specialist at the acute hospital who must approve admissions by the GPs to the community hospital. In many cases, collaboration between community hospitals and larger hospitals or specialists has been described as a means to maximise local provision of services [30]. One example is the re-opening of the maternity unit in Mareeba District Hospital in Queensland, Australia as a midwifery-led model of care described above [54]. The unit is supported by an obstetrician at the base hospital who oversees all emergency care and pregnancy complications.

In many cases, collaboration between the community hospital and other health services is supported by the introduction of new technologies. Examples include a shared electronic health record to help facilitate links between the community hospital and primary care in Norway [45] or a telemedicine link between the community hospital and a larger hospital. Use of telemedicine often involved direct interaction between the specialist and the patient, such as a tele-ophthalmology service in Australia [26,72]. Other examples include the provision of a medical oncology outreach clinic, where oncologists from a larger hospital review patients in the community hospital using video conferencing equipment [55], therapy sessions delivered by videoconference [51], videoconference fracture clinics [33], telepharmacy [73] and remote commenting by radiographers [74]. In northeast Scotland, a minor injuries telemedicine network connects 15 minor injury units in community hospitals to the emergency department at the regional teaching hospital [66]. Patients are seen by trained community hospital nurses, who can seek advice as required from medical staff and consultants based at the teaching hospital emergency department.

### Ownership of community hospitals

Most community hospitals described in studies included in this review are public hospitals, which are the responsibility of local or regional health authorities with regard to funding, management and commissioning of services. However, reflecting the specific system context in different countries, ownership and management may take different forms. For example, an intermediate care department in Trondheim in the north of Norway, was established at a teaching nursing home to provide care for older patients initially admitted to the city acute hospital, but who no longer require acute medical supervision [46]. The goal was to create a new link between specialist care at a general acute hospital and community home care to aid recovery before final discharge of the patient to their own home. Under the Norwegian decentralised model of health care provision, the nursing home falls under the responsibility of the municipality.

One other example is that of a community hospital in Norfolk in the east of England, which is operated as a social enterprise following closure of inpatient beds previously operated by the NHS in 2005 [75]. Additionally, a 28-bed community hospital in Oxford, England, was described earlier. Considered “unfit for purpose”, it was integrated as a unit within a nearby acute tertiary hospital [40].

## Discussion

Based on the published literature of community hospitals in high income countries, we find that community hospitals may provide a wide spectrum of health services, including preventative and primary care, inpatient and outpatient services, medical and surgical care, and acute and chronic care. They are predominantly staffed by GPs and nurses, and may be supported by acute hospital specialists either on a visiting basis or through remote communication. Community hospitals frequently reported strong collaboration with acute hospitals, as well as integration with primary care through a shared workforce.

Our literature search included studies published since 2005, building on a previous review by Heaney et al. [1]. Our findings show many similarities with this earlier review regarding the role of community hospitals within health care provision, and with the types of services offered. We did not identify studies on certain specific services described by Heaney et al., such as cardiac care, however the literature reviewed in the present study reported on a more diverse range of service provision, which are often supported by acute specialists working remotely. We also found evidence of a wider use of telemedicine than described previously. As a scoping review, which aimed to map the evidence on community hospitals; this review did not assess the quality of the evidence, nor did it assess the effectiveness of the different types of community hospitals. We therefore cannot derive any conclusion about which service formulations may be most appropriate. Furthermore, our review focused on published studies, which means that we only capture information on factors that were described by study authors. We do not capture information on community hospitals not reported in the range of sources we considered for the review or literature pertaining to services that may form part of a community hospital, but where the relevant study does not mention the facility itself, for example those examining midwifery units which may be located in community hospitals [76]. Furthermore, we will have found little evidence on topics that are difficult to measure or analyse, a challenge faced by many other reviews of complex care models. These limitations suggest the need for more systematic interrogation of practice in community hospitals through primary research.

**Figure 2:**
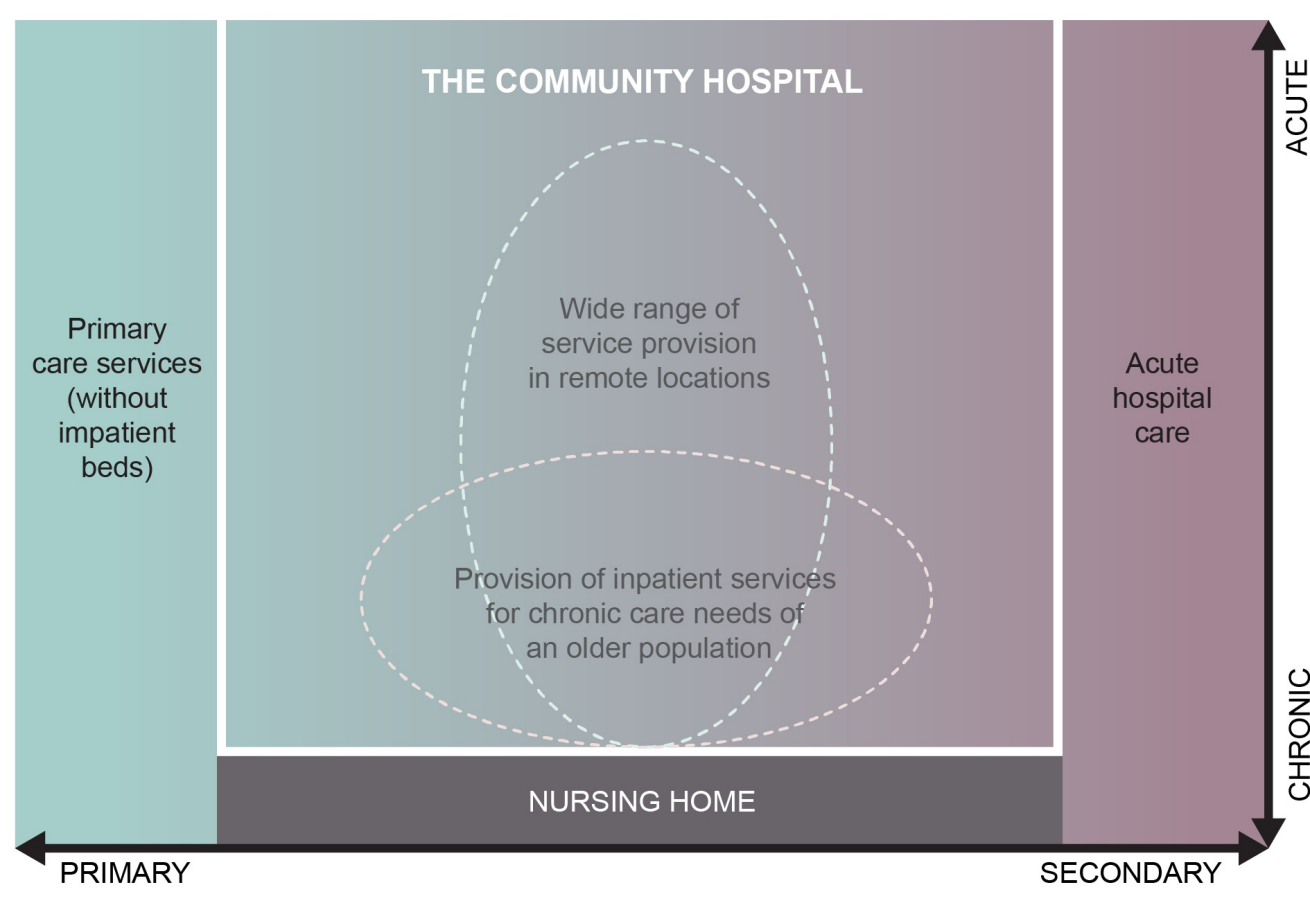
Nature and scope of services provided by CHs.

This review was driven by our *a priori* working definition of community hospitals: those which provide inpatient beds, are led by community-based health professionals, and provide a range of services to a local community, and our findings are thus constrained within these boundaries. However, within this broad definition the review has revealed the wide variety of community hospital models, with notable diversity in the range of services provided and in staffing arrangements to deliver them. This is further summarised in Figure 2, which depicts our understanding of the nature and scope of services provided by community hospitals, based on our review of the evidence. We believe that this figure can provide a helpful way to conceptualise the remit of community hospitals, obviating the need to provide a precise definition for what we have found to be an inherently variable model of service delivery. This conceptualisation sees community hospitals occupying the space between, and to some extent encompassing primary care services, care and nursing home services, and acute hospital care. At the same time, services delivered by community hospitals stretch across acute, chronic and end-of-life care. We found that community hospitals performed many different roles in different contexts, including functioning as an extension of primary care through provision of outpatient services, as an alternative to acute hospital care for low-level inpatient needs and as stepdown facilities from acute care. We also describe examples of community hospitals which focus on the delivery of non-acute inpatient care, such as post-acute care or rehabilitation care for older people, and others, which deliver a wide range of health care to a whole population, often in geographically remote locations where alternative services are not readily available. These potential areas of focus are indicated by the dashed circles in Figure 2.

One feature which was common to most community hospitals was the delivery of care by generalist doctors (GPs) and nurses. Although our initial definition broadly stated that the hospital should be led by “community-based health professionals”, in the majority of examples identified by our review the hospitals were led by GPs or nurses, with only a few exceptions such as non-specialist medical officers in New Zealand, and community geriatricians in England.

Our initial working definition stated that a community hospital should provide inpatient beds, which is in accordance with the WHO definition of a hospital which specifies the provision of inpatient facilities [77]. Therefore, studies which described entities without inpatient beds were not included in our review. However, it is important to note that in England, the Community Hospital Association noted in 2008 that “16 community hospitals no longer have inpatient beds, and they are therefore categorised as Community Resource Centres” [78] and there may be increasing policy emphasis on their provision of outpatient, rather than inpatient, care. This is further reflected in the more recent direction in England, which sees community hospitals expanding their diagnostic and outpatient services. Given this trend, the distinctions between a ‘hospital’ which provides beds and a ‘resource centre’ or ‘enhanced primary care facility’ may be increasingly unhelpful when designing service delivery systems.

One particular feature of community hospitals appears to be their focus on health care provision to reflect local need, responding to geographical and health system contexts. The majority of the community hospitals covered in this review were located in rural settings, while there were also examples of urban community hospitals. However, reviewed studies frequently provided little information on the population or geographical area served by the hospitals described, which limited our ability to explore the degree to which community hospitals adapt or develop in response to local contextual factors.

The evidence reviewed provided many examples for the provision of particular specialist services in community hospital settings, including inpatient and outpatient services, which can be delivered in community hospital settings on a routine or intermittent basis, often with the aid of technological innovations. These findings provide important insights to inform the wider policy debate on shifting care into the community [79]. Joint working arrangements such as visits by travelling surgeons, shared posts across community and acute hospitals, or the use of telemedicine have allowed an increase in the range of services available in community hospitals as well as the level of specialisation of care delivered within community hospitals. Future technological developments allowing medical care to be delivered at a distance may be able to expand the role of community hospitals further and to further address inequalities of health service access.

The studies reviewed highlighted the potential for community hospitals to act as an integrator of services locally. This potential integrative role, together with flexible service development, places the community hospital in a strong position to respond to the current changing health system environment, in particular with regard to challenges posed by the rising number of people with multiple chronic conditions, combined with an ageing population, against a background of constrained resources [9]. Community hospitals may be able to take a key role in new models of care being developed, such as those proposed in England [15], through provision of a ‘community hub’ which already hosts a wide range of services, provides a setting for integration between health and social care organisations, and has strong links with the local community.

It was outside the scope of this study to assess the cost-effectiveness of the provision of services in a community hospital compared with the provision of these services in other settings. Heaney et al. (2006) noted a lack of sufficiently robust evidence on the cost-effectiveness of community hospitals [1], and it will be important to examine in detail the different services provided and the costs and benefits of provision in different settings.

## Conclusion

This scoping review, drawing on literature from 10 high-income countries published since 2005, found that community hospitals operate in a manner which situates them at the boundary of primary care, acute hospital care and nursing home care and that cover the full spectrum of service provision from preventative and primary care, to inpatient surgical or medical care. Services tend to be delivered by generalist doctors and nurses, with specialist physicians visiting occasionally to deliver particular services in some cases. The literature highlighted the potential for community hospitals to respond to different geographical and health system contexts and their integrative role in local service provision which may be particularly important in the design of future models of care delivery, where an emphasis will be placed on integration of patient-centred care from those traditionally situated in different care sectors.
